# Multispectral Raman Differentiation of Malignant Skin Neoplasms In Vitro: Search for Specific Biomarkers and Optimal Wavelengths

**DOI:** 10.3390/ijms241914748

**Published:** 2023-09-29

**Authors:** Elena Rimskaya, Svetlana Shelygina, Alina Timurzieva, Irina Saraeva, Elena Perevedentseva, Nikolay Melnik, Konstantin Kudrin, Dmitry Reshetov, Sergey Kudryashov

**Affiliations:** 1 Lebedev Physical Institute, 119991 Moscow, Russia; rimskaya@lebedev.ru (E.R.); shelyginasn@lebedev.ru (S.S.); a.timurzieva@lebedev.ru (A.T.); saraevain@lebedev.ru (I.S.); perevedencevaev@lebedev.ru (E.P.); melniknn@lebedev.ru (N.M.); k.kudrin@lebedev.ru (K.K.); 2 Semashko National Research Institute of Public Health, 105064 Moscow, Russia; 3 Department of Oncology, Radiotherapy and Reconstructive Surgery, Sechenov First Moscow State Medical University, 119991 Moscow, Russia; 4 Department of Oncology and Radiation Therapy, Evdokimov Moscow State University of Medicine and Dentistry, 127473 Moscow, Russia; reshetov1973@inbox.ru

**Keywords:** basal cell carcinoma, squamous cell carcinoma, skin cancer, confocal Raman and photoluminescence microspectroscopy, optical biopsy, signal processing, diagnostic biomarkers

## Abstract

Confocal scanning Raman and photoluminescence (PL) microspectroscopy is a structure-sensitive optical method that allows the non-invasive analysis of biomarkers in the skin tissue. We used it to perform in vitro diagnostics of different malignant skin neoplasms at several excitation wavelengths (532, 785 and 1064 nm). Distinct spectral differences were noticed in the Raman spectra of basal cell carcinoma (BCC) and squamous cell carcinoma (SCC), compared with healthy skin. Our analysis of Raman/PL spectra at the different excitation wavelengths enabled us to propose two novel wavelength-independent spectral criteria (intensity ratios for 1302 cm^−1^ and 1445 cm^−1^ bands, 1745 cm^−1^ and 1445 cm^−1^ bands), related to the different vibrational “fingerprints” of cell membrane lipids as biomarkers, which was confirmed by the multivariate curve resolution (MCR) technique. These criteria allowed us to differentiate healthy skin from BCC and SCC with sensitivity and specificity higher than 95%, demonstrating high clinical importance in the differential diagnostics of skin tumors.

## 1. Introduction

The morbidity of the population with malignant skin neoplasms represents one of the most common factors among all malignant tumors. Early diagnostics of malignant skin neoplasms, including basal cell carcinoma (BCC) and squamous cell carcinoma (SCC), is an extremely relevant and socially significant problem, as confirmed by the morbidity and mortality rate statistics in the Russian Federation and worldwide [1,2,3].

In the early stages of the pathology’s development, the clinical patterns of the neoplasm are not sufficiently pronounced and differential clarifying diagnostics are needed, which can be handled without additional diagnostic techniques, only by highly qualified medical workers. The final diagnosis is determined with high accuracy with the help of histological studies of surgically removed tissues, which may reduce the treatment efficiency [1,4,5].

The diagnosis of a malignant skin neoplasm at an early stage of development provides a greater likelihood of a favorable treatment outcome, reduces its cost and allows the patient to recover in a shorter time [5,6,7]. However, the existing non-invasive methods (dermatoscopy, thermometry, ultrasound skin scanning, cross-polarization optical coherence tomography, spectroscopy and visualization in the terahertz range [4,5,6,7,8], etc.) and tools widely used in oncology do not provide sufficient accuracy for early diagnostics [5,6,7]. A promising way to solve this problem is the search for new optical methods for the early non-invasive diagnostics and analysis of the human tissues’ structures. One of these methods, which has significant potential to increase the efficiency of the early diagnosis of skin tumors, is confocal scanning Raman and photoluminescence (PL) microspectroscopy, with an excitation wavelength lying in the visible and near-infrared range (e.g., 532, 785, 1064 nm) [9,10,11,12,13,14,15,16,17,18].

Studies have shown that Raman microspectroscopy is sensitive to the basic biochemical variations of cancerous cells at the microscopic level and thus is able to detect specific molecular anomalies (biomarkers) in biological tissues. In combination with the fluorescence effect, it provides enhanced Raman signals from biomolecules with a subsequent assessment of changes in the chemical compositions of biomarkers such as tryptophan, carotenoids, lipids and proteins [19,20,21,22,23,24,25,26]. Meanwhile, the search for novel, accurate Raman spectral criteria, providing high sensitivity and specificity in tumor differentiation, is still in progress.

In this study, we used multi-wavelength (532, 785 and 1064 nm) Raman/PL microspectroscopy to differentiate in vitro samples of healthy skin, BCC and SCC. In particular, we searched for biomarkers and spectral differentiation criteria that allowed the in vitro diagnostics of tumors.

## 2. Results and Discussion

**Figure 1:**
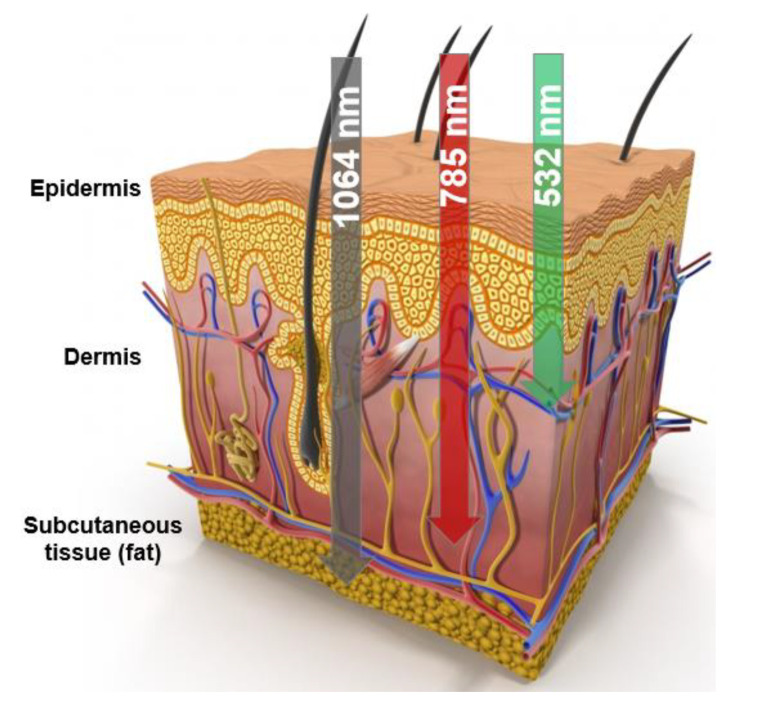
The skin cross-section showing dermal penetration by different wavelengths of laser excitation in the visible–near-infrared range (532, 785 and 1064 nm).

Raman/PL microspectroscopy provides substantial information about the state and morphological structure of a skin neoplasm [18,19,20,21,22]. However, the low signal-to-noise ratio and high level of fluorescence cause some problems in obtaining spectral data with appropriate quality for the further analysis of the Raman bands. Excitation at 532 nm causes a high level of unwanted fluorescence, in contrast to the 785- and 1064-nm wavelengths, which help to minimize the background fluorescence of tissue and may therefore be optimal for the measurement of fresh tissue [10,11,15,27]. The penetration depth of the laser light and the degree of its scattering also depend on the wavelength, as well as on the tissue properties (Figure 1). Therefore, we investigated the effect of the different wavelengths (532, 785 and 1064 nm) of the laser excitation on the Raman spectroscopic manifestations of skin biomarkers.

Comparing the mean Raman/PL spectra of healthy skin (normal skin), BCC and SCC measured at different excitation wavelengths, evident differences in band positions and intensities were obtained. The vibrational assignments for the major healthy skin Raman bands are summarized in Table 1 [4,7,8,11,18,19,20].

### 2.1. Raman/PL Spectra at 532 nm Laser Photoexcitation

**Figure 2:**
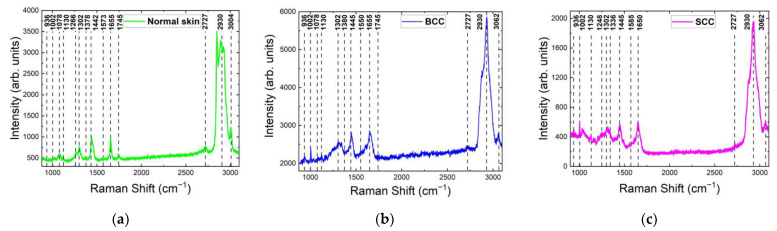
Raman and photoluminescence (PL) spectra of (**a**) healthy skin (normal skin), (**b**) basal cell carcinoma (BCC) and (**c**) squamous cell carcinoma (SCC) at 532 nm laser excitation in 900–3100 cm^−1^ range.

Figure 2 demonstrates examples of registered Raman/PL spectra of healthy skin, BCC and SCC at 532 nm laser excitation in the 900–3100 cm^−1^ range. All skin tissues included in this study had similar major Raman bands (as listed in Table 1 and shown in Figure 2). The bands of the main molecular components, i.e., proteins, lipids and nucleic acids, predominated in the Raman spectra of normal skin, BCC and SCC. The ratio between band intensities reflected the state and morphological structure of tissue on the molecular level and may permit us to search for spectral manifestations of skin biomarkers for differential diagnostic purposes. The major peak positions of the Raman bands for normal skin were at 936, 1002, 1078, 1130, 1266, 1302, 1360, 1442, 1550, 1655 and 1745 cm^−1^. Similarly, the peaks were centered at 936, 1002, 1266, 1302, 1360, 1445, 1510, 1550, 1585, 1655 and 1745 cm^−1^ for BCC and at 936, 1002, 1130, 1248, 1310, 1336, 1445, 1555, 1585 and 1650 cm^−1^ for SCC (see Table 1 for the Raman active components corresponding to these bands).

**Figure 3:**
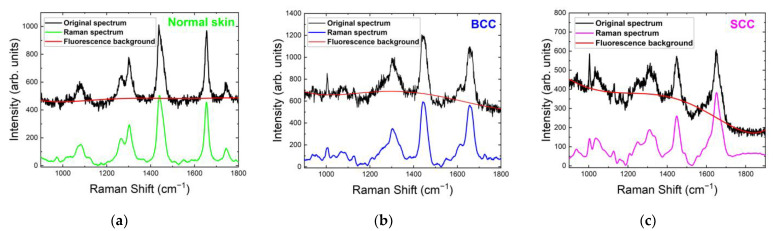
Raman/PL spectra (original spectra), Raman spectra (with background subtracted) and fluorescence background of (**a**) normal skin, (**b**) BCC and (**c**) SCC at 532 nm laser excitation.

The spectra obtained with excitation at 532 nm contained a broad, exponentially decreasing fluorescence curve with prominent Raman peaks, so the most important step in Raman signal processing is the removal of the fluorescence background that is superimposed on the Raman signal. The most commonly used method in biomedical skin tissue measurements is the Vancouver Raman Algorithm, which combines peak removal with a modified polynomial fitting [28,29,30,31,32]. An example of the fluorescence background calculated by the Vancouver Raman Algorithm and the final Raman spectra smoothed by the Savitzky–Golay method are shown in Figure 3.

**Figure 4:**
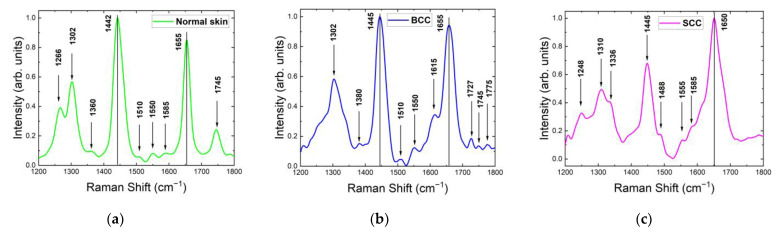
Normalized mean Raman spectra of (**a**) normal skin, (**b**) BCC and (**c**) SCC at 532 nm laser excitation in the range of 1200–1800 cm^−1^.

**Figure 5:**
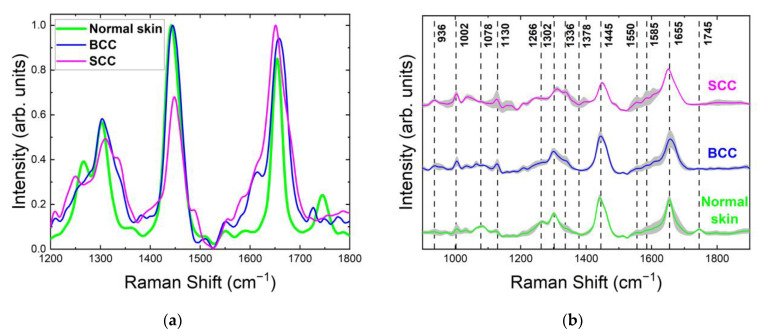
(**a**) Normalized mean Raman spectra with (**b**) standard deviation intervals (solid lines with grey space) of normal skin, BCC and SCC (10–20 independent sites) at 532 nm laser excitation.

For all tested skin samples in the 1200–1800 cm^−1^ range, the major Raman bands appeared within 1260–1340, 1420–1480 and 1620–1700 cm^−1^ (Figure 4). The Raman spectra of normal skin, BCC and SCC shown in Figure 4 were normalized to the maximum intensity of their strongest Raman bands and then averaged. The strongest Raman bands of normal skin, BCC and SCC were located around 1442 cm^−1^, 1445 cm^−1^ and 1650 cm^−1^, respectively. The most significant changes in the Raman spectrum between healthy skin and tumors were observed in the bands 1260–1340 cm^−1^ and 1700–1800 cm^−1^ (Figure 4 and Figure 5a). Interestingly, a double peak was observed for normal skin and SCC in the band 1260–1340 cm^−1^. The existing differences in the intensities of the Raman peaks can be explained by structural tissue disorders during the development of tumors [15]. The amide III band is more prominent in normal skin and SCC, located at 1266 cm^−1^ in normal skin and at 1248 cm^−1^ in SCC, while BCC spectra do not show this band prominently. The band at 1302 cm^−1^, assigned to the CH_2_ vibrations in saturated lipids, is sensitive to the conformational disorder in the cells [23]. It shifts to a higher frequency in SCC and broadens in both BCC and SCC. This increment in the Raman shift indicates an increase in the lipid disorder (the higher content of gauche conformers) [23]. The 1360 cm^−1^ band, possibly originating from tryptophan, narrows in BCC and disappears in SCC. Another tryptophan-related band at 1550 cm^−1^ is visible only in BCC spectra. Lieber et al. [33] have also reported the appearance of this band in BCC, which may be caused by the transport of amino acids. The band at 1488 cm^−1^ was detected only in SCC (Figure 4c), and it may be caused by the intensified cell proliferation that leads to the spatial overcrowding of nucleated cells [34,35]. The band at 1510 cm^−1^ was assigned to carotenoids [36,37,38,39]. Although, in previous works, the carotenoid content in BCC was found to be higher than in normal skin [38], we did not observe such a trend in our work. In addition, the carotenoid content may depend on the individual traits of the patient and may be affected by their lifestyle (smoking, fatigue, illness, etc.) [36]. C=C and composite vibrational bands including the bending (in-plane) N-H and stretching C-N vibrations from the amide II band, located at 1555 cm^−1^ [40], increased in SCC (Figure 4c). This band has been also attributed to tryptophan [41], and the authors observed its increase in BCC and SCC. Phe is also presumably represented by the band at 1585 cm^−1^ [11,19,21,24]. The band at 1615 cm^−1^ may be assigned to tyrosine, and its height increases in BCC and decreases in SCC. Amide I (1655 cm^−1^) shifts towards a lower frequency in BCC and SCC. The reduction in the band intensity in BCC was previously reported by Gniadecka et al. [42]. The accumulation of large numbers of SCC tumor cells results in enlarged nucleoli and shrunken cytoplasm, leading to a higher nucleo-cytoplasmic ratio [43]. The band at 1727 cm^−1^, assigned to amino acids [26], exhibits an increase in intensity in BCC, which may be caused by the transport of amino acids [19,21]. It is noteworthy that initially one band at 1745 cm^−1^ in normal skin was split into two bands with reduced intensity in tumors, which was attributed to ester vibrations in lipids [24]. Other bands in the ~1800 cm^−1^ region were also assigned to lipids, and their intensity decreased in both BCC and SCC.

As a result, the differences between the Raman spectra of normal skin and tumors (BCC, SCC) may be associated with an increase in the concentration of nucleic acids and a change in the structure of proteins in the cells of the tumor, as well as with a decrease in the intensity of the lipid-specific bands.

The normalized mean spectra (color curves) with standard deviation intervals (grey shading) shown in Figure 5b are representative of the robustness of the spectral features. A wider variety of spectral intensities in particular bands indicates higher diversity in the individual properties of samples from different patients and their distribution across each sample. For the Raman spectra of normal skin, a significant deviation range was observed around 900–1050 cm^−1^ (C–C and P=O stretching, ring breathing), 1190–1260 cm^−1^ (amide III), 1336 cm^−1^ (CH_2_ wagging), 1520–1740 cm^−1^ (amide I, C=C stretching) and 1800 cm^−1^ (C=O stretching). By contrast, the Raman spectra of SCC demonstrated greater variance in the regions of 1080–1200, 1340–1400 and 1520–1630 cm^−1^ and similar or lower variability for vibration bands at 940–1000, 1336 and 1445 cm^−1^, having a significant contribution of the main tissue constituents. A noticeable discrepancy between the BCC spectra was seen around 930–1010 cm^−1^, 1080–1140 cm^−1^, 1302 cm^−1^, and in the region of 1520–1700 cm^−1^, where the deviation was greater compared to normal skin.

### 2.2. Raman/PL Spectra at 785 nm Laser Photoexcitation

Raman/PL microspectroscopy at 785 nm is a non-destructive measuring technique commonly used for the analysis of biological tissues, because this wavelength helps to minimize the fluorescence background of tissue and may be optimal for the measurement of characteristic Raman features [33,41]. Moreover, a sufficient signal-to-noise ratio can be achieved with a shorter exposure time compared to Raman/PL measurements at the 532 nm wavelength. Excitation at 785 nm leads to a significant enhancement in the Raman/PL intensity of specific vibration modes relating to chromophores that absorb near this excitation wavelength.

**Figure 6:**
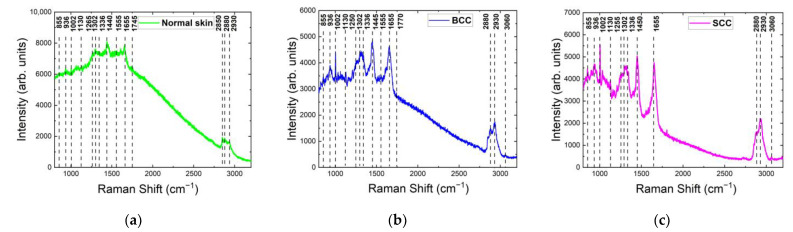
Raman/PL spectra of (**a**) normal skin, (**b**) BCC and (**c**) SCC at 785 nm laser excitation in 900–3100 cm^−1^ range.

The Raman/PL spectra of normal skin (healthy), BCC and SCC were registered at 785 nm laser excitation in the 900–3100 cm^−1^ range. The acquired data indicated that the analyzed skin tissues, when excited by a laser with a wavelength of 785 nm, had similar major Raman bands compared with the data obtained at 532 nm excitation (Table 1, Figure 6). In addition to these Raman bands of common skin tissue constituents, the Raman spectra at 785 nm possessed intense resonance bands of tissue chromophores (carotenoids, etc.) [11,38,39]. The major Raman bands for normal skin were located at 855, 936, 1002, 1130, 1265, 1302, 1336, 1360, 1440, 1525, 1555, 1655 and 1745 cm^−1^. The spectra for tumors indicated the presence of the main bands at 855, 936, 1002, 1250, 1302, 1336, 1385, 1445, 1525, 1555, 1585, 1655 and 1770 cm^−1^ for BCC and at 855, 936, 1002, 1130, 1255, 1302, 1336, 1450 and 1655 cm^−1^ for SCC (see Table 1 for the corresponding Raman active components).

**Figure 7:**
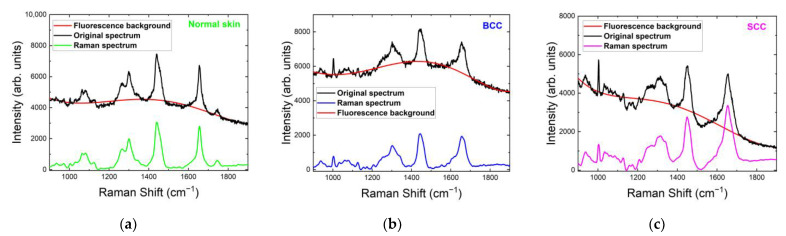
Raman/PL spectra (original spectra), Raman spectra (with background subtracted) and fluorescence background of (**a**) normal skin, (**b**) BCC and (**c**) SCC at 785 nm laser excitation.

The registered Raman/PL spectra (original spectra), Raman spectra without the fluorescence background (Raman spectra) and the fluorescence background of normal skin, BCC and SCC at 785 nm laser excitation are presented in Figure 7.

**Figure 8:**
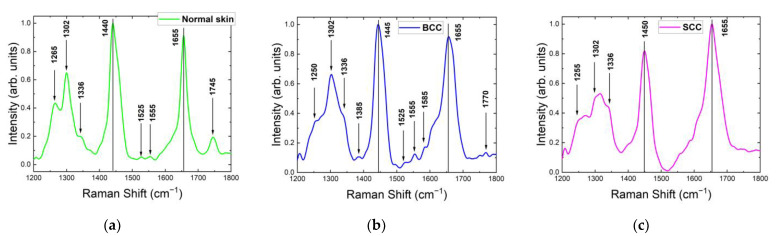
Normalized mean Raman spectra of (**a**) normal skin, (**b**) BCC and (**c**) SCC in the range of 1200–1800 cm^−1^ at 785 nm laser excitation.

**Figure 9:**
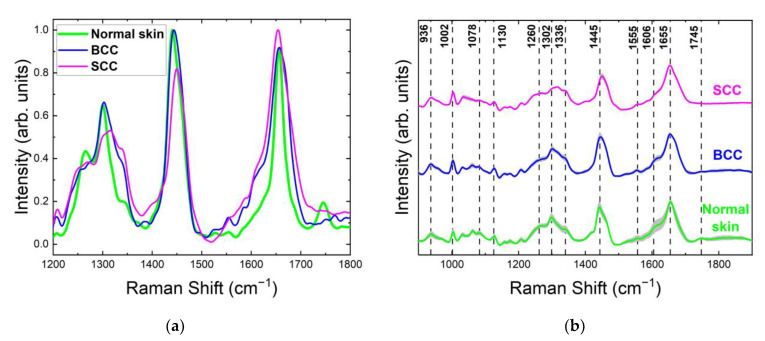
(**a**) Normalized mean Raman spectra with (**b**) standard deviation intervals (solid lines with grey space) of normal skin, BCC and SCC (10–20 independent sites) at 785 nm laser excitation.

The normalized mean Raman spectra for different skin samples in this study are presented in Figure 8. The major Raman bands for all skin samples were located within 1245–1340, 1390–1480 and 1590–1700 cm^−1^. The strongest Raman band of normal skin, BCC and SCC was located around 1440 cm^−1^, 1445 cm^−1^ and 1655 cm^−1^, respectively. The most significant changes in the Raman spectrum between healthy skin and tumors were observed in the bands 1245–1340 cm^−1^ and 1700–1800 cm^−1^ (Figure 8 and Figure 9a). As with 532 nm, the Raman spectra of normal skin and SCC had a double peak in the band 1245–1340 cm^−1^. Interestingly, at 785 nm, a double peak in this band was also seen in the Raman spectra of BCC. The amide III band was more expressed in normal skin and located at 1265 cm^−1^, unlike BCC and SCC, which had weak bands at 1255 and 1250 cm^−1^, respectively. CH_2_ vibrations in saturated lipids (1302 cm^−1^) were observed in all Raman spectra of skin samples at 785 nm, unlike the Raman spectra of SCC at 532 nm (1310 cm^−1^). The vibration band at 1336 cm^−1^ (CH deformation in proteins) narrowed in both BCC and SCC, with its intensity increasing. The narrowing of the bands may result from the rigidification of the nucleic acids [44]. The 1360 cm^−1^ band, which is visible at 532 nm in normal skin and BCC, is missing at 785 nm in all tissues; however, another tryptophan-related band at 1555 cm^−1^ is visible in all Raman spectra at this wavelength. The carotenoid (band at 1525 cm^−1^) content in BCC was found to be higher than in normal skin [38]; in SCC, it is missing. Phe is represented by the band at 1585 cm^−1^ in BCC and SCC [26]. The band intensity at 1655 cm^−1^ in BCC was unchanged relative to normal skin, but in SCC, it increased by almost 10%. The initially one band at 1745 cm^−1^ in normal skin was split into two bands each in BCC and SCC with decreased intensity, similarly to the case at 532 nm [12,15]. The intensity of other bands in the ~1800 cm^−1^ region decreased in both BCC and SCC.

For the normalized mean Raman spectra of normal skin, significant variability was observed around 900–1010 cm^−1^, 1330−1340 cm^−1^, 1445 cm^−1^, 1550–1650 cm^−1^ and 1800 cm^−1^, having a significant contribution of the main tissue constituents. By contrast, the Raman spectra of SCC and BCC include regions demonstrating lower variability in comparison with normal skin: 1330−1340 cm^−1^, 1550–1650 cm^−1^ and 1800 cm^−1^ (Figure 9b).

### 2.3. Raman/PL Spectra at 1064 nm Laser Photoexcitation

**Figure 10:**
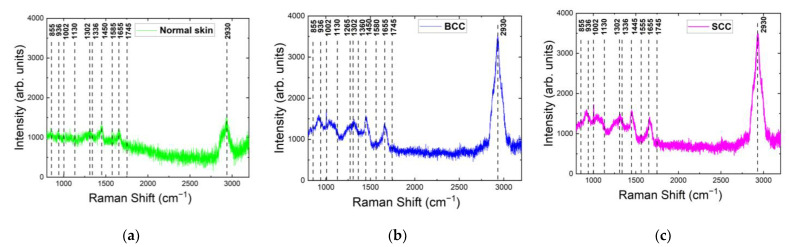
Raman/PL spectra of (**a**) normal skin, (**b**) BCC and (**c**) SCC at 1064 nm laser excitation in 900–3100 cm^−1^ range.

**Figure 11:**
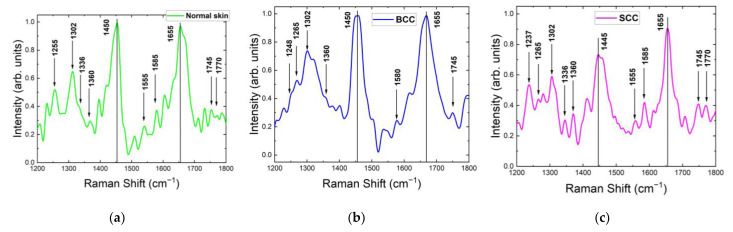
Normalized mean Raman spectra of (**a**) normal skin, (**b**) BCC and (**c**) SCC in the range of 1200–1800 cm^−1^ at 1064 nm laser excitation.

The measuring of Raman/PL spectra at 1064 nm has been widely used for the investigation of various human tissues, including tumors, without destruction (thermal damage, photo damage) [12,30,32,33,34,35,36], because this wavelength is non-destructive and non-mutagenic. The Raman/PL spectra of normal skin, BCC and SCC were registered at 1064 nm laser excitation in the 900–3100 cm^−1^ range. However, due to the low signal-to-noise ratio, it was quite difficult to isolate the main bands of the Raman spectra. The skin tissues at the 1064 nm wavelength shared the same major Raman bands compared with 532 nm and 785 nm (Table 1, Figure 10 and Figure 11). The major Raman bands for normal skin were located at 855, 936, 1002, 1130, 1255, 1302, 1336, 1360, 1450, 1555, 1585, 1655, 1745 and 1770 cm^−1^. Those for BCC were centered at 855, 936, 1002, 1248, 1265, 1302, 1360, 1450, 1580, 1655 and 1745 cm^−1^, and those for SCC were centered at 855, 936, 1002, 1130, 1237, 1265, 1302, 1336, 1360, 1445, 1555, 1585, 1655, 1745 and 1770 cm^−1^ (the Raman active components of these bands can be seen in Table 1).

**Figure 12:**
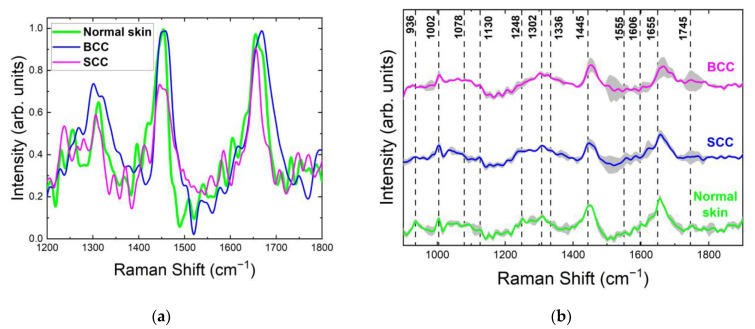
(**a**) Normalized mean Raman spectra with (**b**) standard deviation intervals (solid lines with grey space) of normal skin, BCC and SCC (10–20 independent sites) at 1064 nm laser excitation.

Figure 11 demonstrates the normalized mean Raman spectra of normal skin, BCC and SCC in the range 1200–1800 cm^−1^. The major Raman bands for all skin samples are located within 1210–1350, 1400–1490 and 1610–1700 cm^−1^. The strongest Raman band at the 1064 nm wavelength of normal skin and BCC is located around 1450 cm^−1^ and that of SCC at 1655 cm^−1^, as well as at the 532 and 785 nm wavelengths. The most significant changes in the Raman spectrum between healthy skin and tumors were observed in the bands 1210–1370 cm^−1^ and 1700–1800 cm^−1^ (Figure 11 and Figure 12a). The Raman spectra of normal skin and SCC had a strongly pronounced double peak in the band 1220–1330 cm^−1^. Moreover, both at 785 and 1064 nm, a double peak in this band was also seen in the Raman spectra of BCC. The amide III band was more expressed in normal skin, unlike BCC and SCC. The band at 1302 cm^−1^ was well observed in all Raman spectra of the skin samples. The vibration band at 1336 cm^−1^ was clearly visible in SCC, but it was not fully distinguishable in normal skin data and was completely absent in BCC. At the 1064 nm wavelength, all skin tissues had a band at 1360 cm^−1^ (tryptophan). Interestingly, this band is visible at 532 nm in normal skin and BCC, but is missing at 785 nm in all tissues. However, another tryptophan-related band at 1555 cm^−1^ is visible only in the Raman spectra of normal skin and SCC. The band at 1525 cm^−1^ is missing in all skin tissues at this wavelength. In all tissues, Phe is presented. C=O stretching (1745 cm^−1^) in SCC increased, unlike in BCC. The increase in the lipid bands’ intensity (near 1800 cm^−1^) indicated changes in the substances in this region, which may have been due to the presence of cancer cells.

The normalized mean Raman spectra of SCC and BCC include regions demonstrating lower variability in comparison with normal skin: 1445 cm^−1^ and 1480–1800 cm^−1^ (Figure 12b).

### 2.4. Wavelength-Independent Spectral Criteria for Differentiating Diagnostics

Significant differences in the Raman spectra of healthy skin and tumors make it possible to differentiate the types of these tissues using Raman/PL microspectroscopy. Thus, we have developed a method for the determination of various skin tumors based on spectral criteria related to the ratio between the spectral bands of skin biomarkers [8,42,45,46,47]. Since combining the Raman spectra and the fluorescence background has been shown to improve the sensitivity and specificity of tumor detection, we calculated the criteria for the pure Raman spectra and the spectra without background subtraction and compared the results [8,10,15,29]. The band ratios $I_{1302/1445}$ and $I_{1745/1445}$ were determined as the ratio of the maximum Raman intensity in the 1298–1304 cm^−1^ and 1740–1750 cm^−1^ spectral bands, respectively, to the maximum Raman intensity in the 1440–1450 cm^−1^ band (1), to demonstrate spectral changes for lipids:

(1) $$I_{1302/1445}=\frac{I_{1302}}{I_{1445}}\;\mathrm{and}\;I_{1745/1445}=\frac{I_{1745}}{I_{1445}}.$$

These spectral bands have been chosen according to the normalized mean Raman spectra of normal skin (Figure 5, Figure 9 and Figure 12). The ratio of $I_{1745/1445}$ plotted vs. $I_{1302/1445}$ shows the distinctive separation of clusters for normal skin, BCC and SCC. The band at 1445 cm^−1^, assigned to the CH_2_ deformations of proteins and lipids, is relatively conformation-insensitive, being a characteristic band of typical Raman spectra for both tumors and healthy skin, and therefore it has been proposed as an intensity standard [45,46]. The band at 1302 cm^−1^ was assigned to the twisting deformation of the CH_2_ methylene groups of intracellular lipid acyl; hence, the $I_{1302/1445}$ ratio represents the relative lipid content [42,47]. The high intensity band at 1745 cm^−1^ (ester vibrations [48]) appears to be a diagnostic band for oxidative stress, inflammation and disease formation [17], in which there are significant changes in the Raman spectra. These combinations proved to be the most valuable for the discrimination of normal skin and tumors.

The classification performance was evaluated using a cross-validation scheme in MATLAB Classification Learner (R2022b, MathWorks, Natick, MA, USA) and represented by confusion matrices [49] and ROC (receiver operating characteristic) curves [15,31]. This allowed us to assess the similarities and differences between the skin samples, as well as to separate them using discriminant analysis based on fitting Gaussian distributions. We compared the performance of linear and quadratic discriminant analysis and characterized their efficiency in terms of sensitivity and specificity, as given below.

#### 2.4.1. Differentiation at 532 nm Laser Photoexcitation

**Figure 13:**
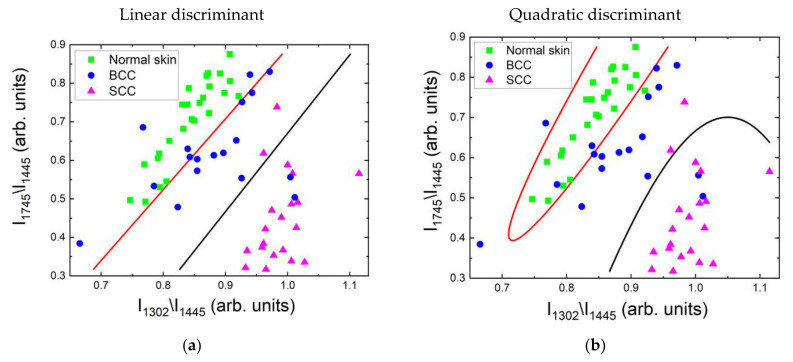

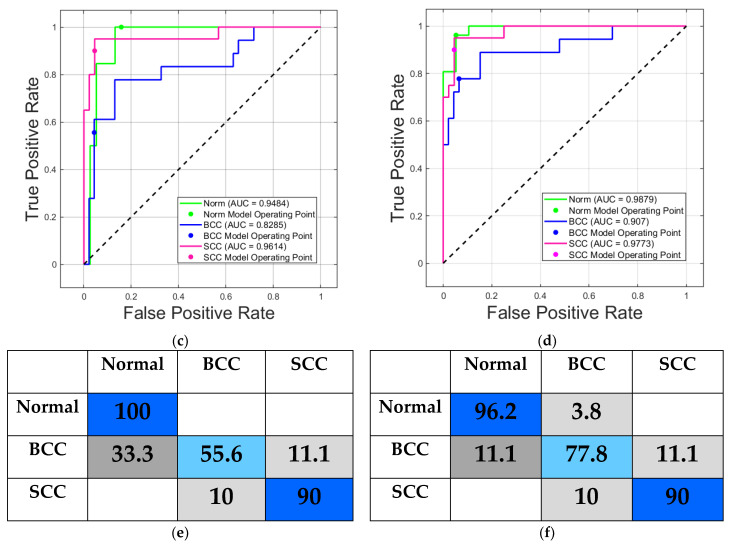
Classification of normal skin, BCC and SCC samples in vitro at 532 nm laser excitation by means of Raman band ratios using linear and quadratic discriminant analysis. (**a**,**b**) Band ratios for determination of various skin tumors: the red line separates normal skin from BCC and SCC, the black line BCC from SCC; (**c**,**d**) ROC curves with AUC values for normal skin, BCC and SCC; (**e**,**f**) confusion matrix of classification in percent for all data (Raman spectra with fluorescent background).

Figure 13a,b show the joint distribution of the two selected spectral criteria and the calculated boundaries between normal skin and BCC (red line) and between BCC and SCC (black line). The ROC curves for the three classes of skin tissue (Figure 13c,d) visualize the true positive rate against the false positive rate for each class. Moreover, ROC AUC (area under curve) scores were calculated as a performance measure. Thus, the ROC AUC values for the linear discriminant analysis of normal skin, BCC and SCC were 0.95, 0.83 and 0.96, respectively; for quadratic discriminant analysis, they were 0.99, 0.91 and 0.98. The classification rate using linear and quadratic discriminant analysis of normal skin was excellent, at 100% and 96.2%, respectively; that of SCC was 90% in both cases, which is similar to the result for dermatology experts. It can be seen that the clusters of normal skin and BCC partially overlap in Figure 13a,e, and thus the separation of BCC and normal skin tissue by a linear boundary is difficult (the classification rate was 55.6%), but classification is possible with a rate of 77.8% using quadratic discriminant analysis (Figure 13b,f).

From an expert point of view, it is interesting to look at the classification accuracy of all tumors versus normal skin. The sensitivity and specificity for the linear discriminant were 100% and 84%, respectively, and the corresponding values for the quadratic discriminant analysis were 96.2% and 94.7%, which is a very good result for the differential diagnostics of tumors.

#### 2.4.2. Differentiation at 785 nm Laser Photoexcitation

**Figure 14:**
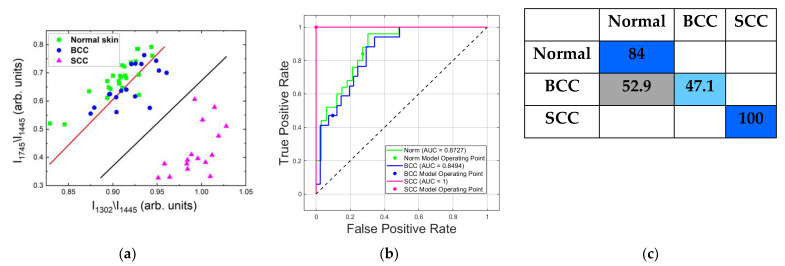
Classification of normal skin, BCC and SCC samples in vitro at 785 nm laser excitation by means of Raman band ratios using linear discriminant analysis. (**a**) Band ratios for determination of various skin tumors: the red line separates normal skin from BCC and SCC, and the black line BCC from SCC; (**b**) ROC curves with AUC values for normal skin, BCC and SCC; (**c**) confusion matrix of classification in percent for all data (Raman spectra with fluorescent background).

Raman/PL microspectroscopy at 785 nm makes it possible to differentiate healthy skin and tumors, especially SCC. In Figure 9, it can be seen that normal skin, BCC and SCC showed significant differences in the Raman spectra. Using two selected spectral criteria allowed us to evaluate the similarities and differences between the skin samples, as well as to classify them using linear discriminant analysis (Figure 14). Figure 14a shows the division into three classes: normal skin, BCC and SCC. The red line demonstrates the separation of normal skin from BCC, and the black line BCC from SCC. Moreover, an ROC curve was constructed to visualize the classification of these classes and ROC AUC scores were also calculated (Figure 14b). The ROC AUC scores for the linear discriminant analysis of normal skin, BCC and SCC were 0.87, 0.85 and 1, respectively. The classification rate of SCC was excellent, at 100%, which is better than its classification at the 532 nm wavelength. The results of classification demonstrated that SCC can be clearly separated from normal skin and BCC. The classification rates of normal skin and BCC were 84% and 47.1%, respectively. The areas of normal skin and BCC strongly overlapped, and thus the separation of BCC and normal skin tissue was very difficult (Figure 14a). The sensitivity and specificity were 84% and 72.7%, respectively, using linear discriminant analysis, which allowed us to differentiate the tumors. The sensitivity and specificity of the classification method at the 785 nm wavelength was significantly lower than at the 532 nm wavelength.

#### 2.4.3. Differentiation at 1064 nm Laser Photoexcitation

**Figure 15:**
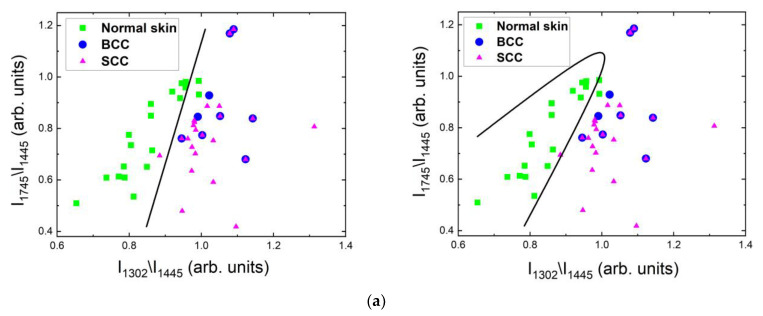

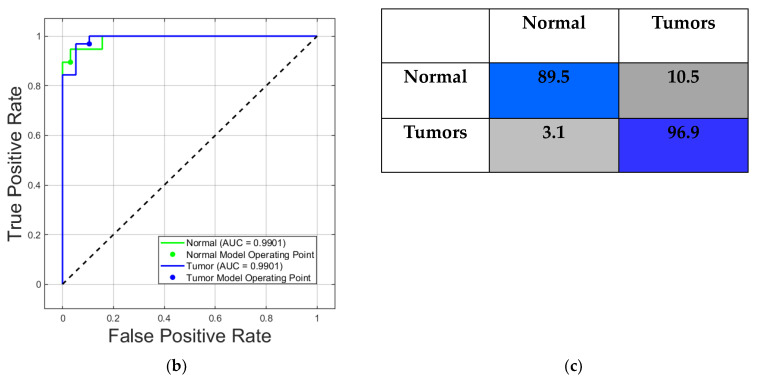
Classification of normal skin, BCC and SCC samples in vitro at 1064 nm laser excitation by means of Raman band ratios using linear and quadratic discriminant analysis. (**a**) Band ratios for determination of various skin tumors: the black line separates normal skin from all tumors (BCC together with SCC); (**b**) ROC curves with AUC values for normal skin and tumors; (**c**) confusion matrix of classification in percent for all data (Raman spectra with fluorescent background).

Raman/PL microspectroscopy at 1064 nm makes it possible to exclusively differentiate healthy skin from tumors. In Figure 15a,b, it can be seen that BCC and SCC had no significant differences in the Raman spectra; it is possible to separate only normal skin. The ratios of the Raman band intensity at 1302 cm^−1^ and 1745 cm^−1^ to 1445 cm^−1^ were calculated for normal skin and tumors (BCC together with SCC). Using linear and quadratic discriminant analysis (Figure 15), it was possible to separate all data into only two classes: normal skin and tumors (the black line demonstrates the separation of normal skin from BCC together with SCC). The ROC AUC scores for the linear and quadratic discriminant analysis of normal skin and tumors were 0.99 in both cases (Figure 15c). The areas of SCC and BCC not only overlapped, but the values in them were the same, and thus the separation of BCC and SCC was very difficult (Figure 15a,b). The sensitivity and specificity were 89.5% and 96.9%, respectively, using both for linear and quadratic discriminant analysis, which allowed us differentiate tumors from healthy skin (Figure 15d).

### 2.5. Multivariate Curve Resolution Analysis for Differentiating Diagnostics

We compared the results obtained by the method using the proposed spectral criteria with the results of multivariate curve resolution (MCR) analysis based on a non-negative matrix factorization (NNMF) algorithm [20,50,51,52,53,54]. Here, we used a MATLAB implementation of the algorithm, which uses an alternating least squares (ALS) method.

The advantage of this analysis is the ability to interpret the main components into which the original Raman/PL spectra are decomposed. The decomposition reveals the spectra of the substances constituting the skin sample and their contributions to the Ra-man/PL spectrum, which are related to their concentrations. The results depend on the chosen number of components; thus, Feng et al. [20] reported that using eight components is optimal for the analysis of skin constituents in samples of normal skin and tumors. In our study, the MCR-ALS analysis was performed for the Raman/PL spectra (Raman spectra with fluorescent background) of normal skin, BCC and SCC. The optimal number of components was also determined to be eight at 532 and 785 nm laser excitation. For the spectra acquired with 1064 nm excitation, decomposition into seven components gave more stable results with smaller residuals, which depended on the characteristics of the original spectra.

#### 2.5.1. Differentiation at 532 nm Laser Photoexcitation

**Figure 16:**
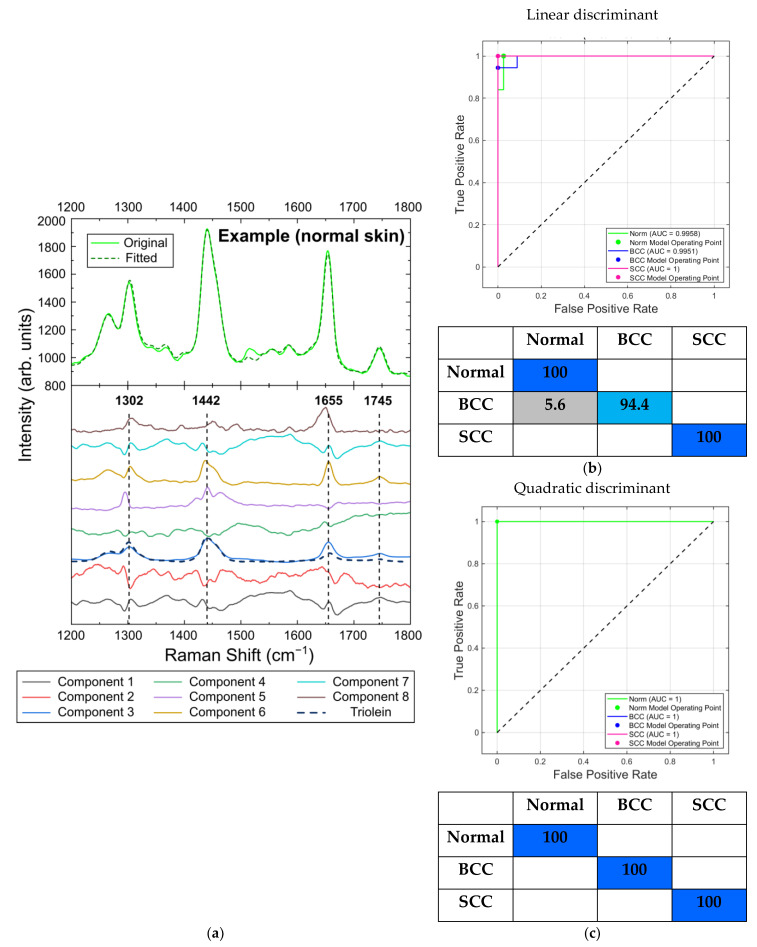
(**a**) Eight components obtained by MCR-ALS analysis of in vitro Raman/PL spectra (Raman spectra with fluorescent background) of skin neoplasms at 532 nm laser excitation (lower part; dashed line denotes the spectra of triolein as given in [20])—original and fitted example spectra of normal skin sample (upper part); ROC curves with AUC values and confusion matrices (classification rate in percent) for normal skin and tumors using (**b**) linear and (**c**) quadratic discriminant analysis.

Figure 16a shows the eight components produced by the MCR-ALS method for the Raman/PL spectra obtained at a 532 nm excitation wavelength and examples of the original and fitted spectra for one of the normal skin samples. The Raman bands used for the selection of the spectral criteria in the previous section are shown by dashed lines, so that one can clearly distinguish them in the spectra of component 3. A comparison with the results obtained by Feng et al. [20] shows the significant similarity of this spectrum to the spectrum of triolein in the range of 1200 to 1800 cm^−1^, with the major Raman bands at 1302, 1442, 1655 and 1745 cm^−1^ (Figure 16a, dashed line). As mentioned before, these bands are associated with lipids: the band at 1302 cm^−1^ is attributed to CH_2_ vibrations in saturated lipids, that at 1442 cm^−1^ to the CH_2_ deformations of lipids, that at 1655 cm^−1^ to C=C stretching amide I vibration and that at 1745 cm^−1^ to ester vibrations in lipids [23,24,42,47,48]. Lipids are the main components of the skin tissue, which is why they contribute significantly to the skin spectrum and can be identified by MCR-ALS analysis in the presence of a fluorescence background. The major components of sebaceous lipids are triglycerides (triolein), which are abundant in subcutaneous fat [20,55]. We observed that the concentration of component 3 in the BCC (6%) and SCC (5%) samples was lower compared to its concentration in normal skin (16%). This result is in accordance with the decrease in the concentration of triolein in the BCC and SCC samples reported in [20], which is caused by the thickening epidermis during the progression of a malignancy, and it justifies the choice of the lipid-related spectral bands for the calculation of the spectral criteria in the previous section.

As before, the classification performance was evaluated using MATLAB Classification Learner and represented by confusion matrices and ROC curves. We compared the performance of linear and quadratic discriminant analysis and characterized their efficiency in terms of sensitivity and specificity. We should note that we did not have sufficient training and testing data sets because of the small number of samples, so the results given below should be considered only as a confirmation of the applicability of the MCR-ALS decomposition and discriminant analysis for the differentiation of tumors in our set of samples.

Thus, the ROC AUC scores for the linear discriminant analysis of normal skin, BCC and SCC were 0.99, 0.99 and 1, respectively; the AUCs for quadratic discriminant analysis were equal to 1 for all skin tissues (Figure 16b,c) when discriminated using all eight components. The sensitivity and specificity for the linear discriminant analysis were 100% and 97%, respectively, and the corresponding values for the quadratic discriminant analysis were 100% in both cases.

#### 2.5.2. Differentiation at 785 nm Laser Photoexcitation

**Figure 17:**
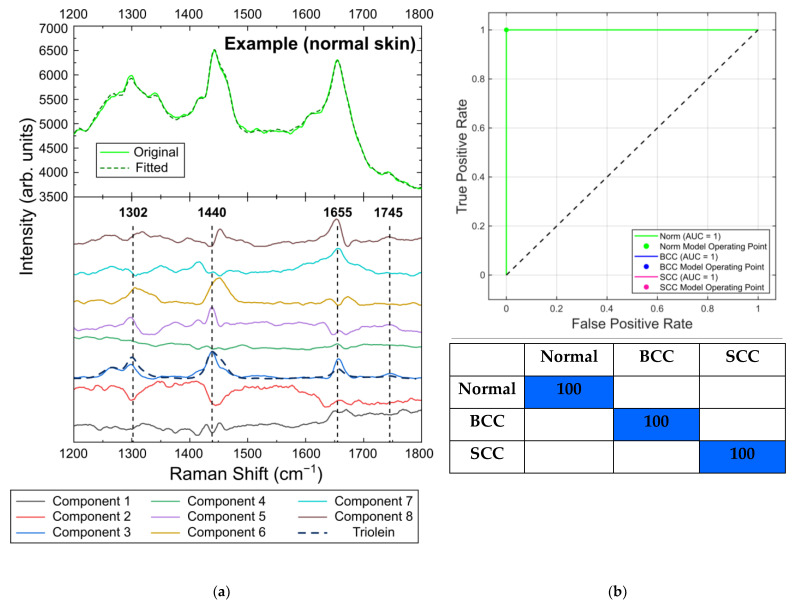
(**a**) Eight components obtained by MCR-ALS analysis of in vitro Raman/PL spectra of skin neoplasms at 785 nm laser excitation (lower part; dashed line denotes the spectra of triolein as given in [20])—original and fitted example spectra of normal skin sample (upper part); (**b**) ROC curves with AUC values and confusion matrices (classification rate in percent) for normal skin and tumors using linear and quadratic discriminant analysis.

Figure 17a demonstrates the results of the MCR-ALS analysis for Raman/PL spectra at 785 nm laser excitation. Similarly to the data obtained at 532 nm, the spectrum of component 3 can be associated with the spectrum of triolein [20] in the range of 1200 to 1800 cm^−1^, with the major Raman bands at 1302, 1440, 1655 and 1745 cm^−1^ (Figure 17a, dashed line). The calculated concentration of component 3 in BCC (10%) and SCC (7%) samples was also lower than the concentration in normal skin (16%); therefore, the conclusion about the optimality of using the lipid-attributed Raman bands at 532 nm remains relevant for the 785 nm wavelength.

The results of linear and quadratic discriminant analysis demonstrate higher ROC AUC values (Figure 17b) equal to 1 for all skin tissues. The classification rate for normal skin, BCC and SCC was 100% in all cases, as well as the sensitivity and specificity.

#### 2.5.3. Differentiation at 1064 nm Laser Photoexcitation

**Figure 18:**
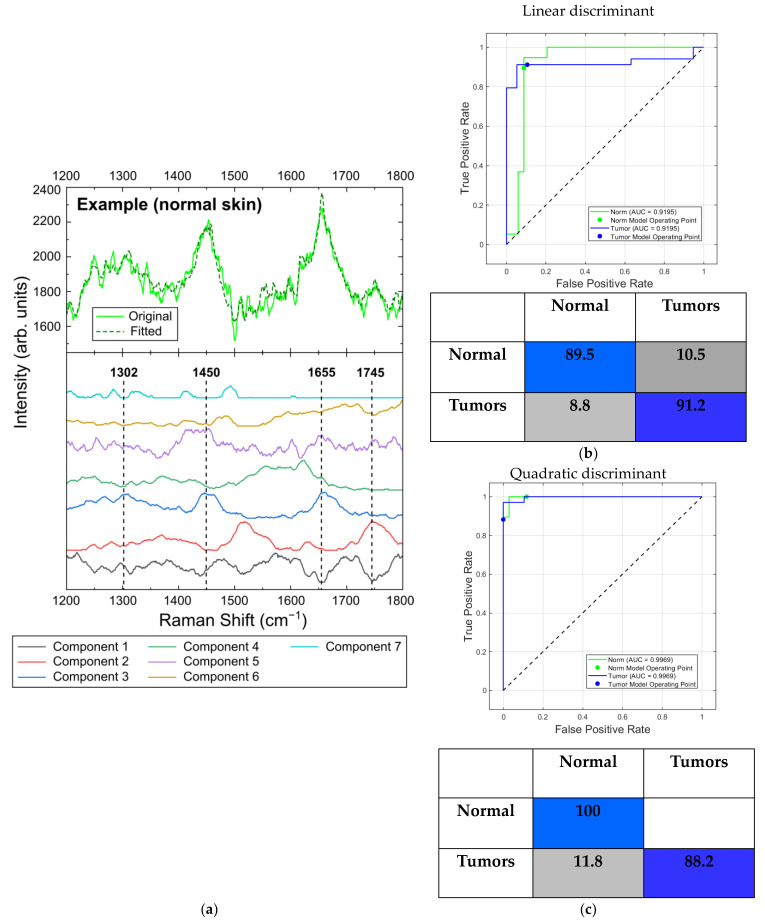
(**a**) Seven components obtained by MCR-ALS analysis of in vitro Raman/PL spectra of skin neoplasms at 1064 nm laser excitation (lower part)—original and fitted example spectra of normal skin sample (upper part); ROC curves with AUC values and confusion matrices (classification rate in percent) for normal skin and tumors using (**b**) linear discriminant and (**c**) quadratic discriminant analysis.

The spectra of seven components calculated using the MCR-ALS technique for Ra-man/PL spectra at 1064 nm laser excitation are presented in Figure 18a. Since the low signal-to-noise ratio and high level of fluorescence cause problems in obtaining spectral data at this wavelength, we cannot directly associate one of the obtained components with the spectrum of triolein [20,55]. However, the spectra of components 2 and 3 include major Raman bands of lipids located at 1302, 1450, 1655 and 1745 cm^−1^. Similar to the results for the 532 nm and 785 nm wavelengths, the calculated concentration of component 3 in BCC (7%) and SCC (6%) was lower relative to its concentration in normal skin (20%).

The ROC AUC scores of both normal skin and tumors were 0.92 and 0.99 for linear and quadratic discriminant analysis, respectively, when using all seven components. The sensitivity and specificity for the linear (89.5% and 91.2%) and quadratic (100% and 88.2%) discriminant analyses were sufficient for the differentiation of the tumors from healthy skin (Figure 18b,c).

## 3. Materials and Methods

### 3.1. Preparation of the Samples

Fresh skin tissue specimens were collected during routine surgical procedures from patients, and these specimens were placed in hermetically sealed cuvettes (each cuvette was marked with a unique number) with physiological saline. On the day of the operation, immediately after the removal of tumor tissues, small tissue fragments up to 3 × 3 mm in size were separated from the samples, which included a tumor area within the intact tissue surrounding it. The material for research was taken in such a way as not to distort the subsequent histological examination of tissues after their removal. No more than 1.5 h passed from the moment that the samples were taken to the start of the spectra acquisition. In this study, we analyzed Raman/PL spectra, collected by scanning 7 BCC, 5 SCC (for each tumor sample, a signal from the surrounding healthy tissue was also recorded) and 5 samples of healthy skin from different patients (approximately equal numbers of men and women 30–60 years old). According to the microscopic biopsy, all BCC and SCC cases were classified as early-stage tumors (grade of cancer: G1 (well differentiated) and G2 (moderately differentiated)).

### 3.2. Confocal Scanning Photoluminescence and Raman Microspectroscopy System

Raman/PL spectra of the samples were acquired by 2D scanning confocal Raman/PL microspectroscopy, using Renishaw “inVia Basis” (inVia InSpect, Renishaw, London, UK) and Confotec MR520 (SOL instruments, Minsk, Belarus) microscope–spectrometers, at 532, 785 and 1064 nm laser excitation wavelengths, at room temperature (25 °C). The excitation laser beam was focused on the sample surface by a 50× objective lens (N Plan 50/0.50 Leica, Germany), and the backward Raman/PL radiation was collected by the same lens. All registered Raman/PL spectra at 532 nm were obtained with a 2 s exposure time; those at 785 and 1064 nm were obtained with a time of 10 s. The power of the laser radiation in the sample plane at 532 nm was less than 20 mW, that at 785 nm was 45 mW and that at 1064 nm was 150 mW. To cover the maximal sample area, the Raman/PL spectra at the different wavelengths (532, 785 and 1064 nm) of laser excitation were obtained from 10–20 independent sites of healthy skin and tumor samples.

### 3.3. Raman Data Processing

The measured Raman signal in the 500–1200 cm^−1^ range was rather weak; therefore, this range was mainly used for the analysis of the fluorescence skin features. In the 1200–1800 cm^−1^ region, a smaller fluorescence contribution was observed, which made it possible to identify Raman bands in the registered signal. The fluorescence background in the 900–1900 cm^−1^ range was subtracted from the original spectra based on the Vancouver Algorithm, which is an improved multipolymodal baseline removal method optimized for fluorescence from biomedical samples. Each spectrum was preprocessed with smoothing by the Savitzky–Golay method. Then, all the Raman spectra with removed fluorescence in the range of 900 cm^−1^ to 1800 cm^−1^ were normalized by the maximum intensity peak in this region because different groups of patients have different concentrations of substances in their tissues.

## 4. Conclusions

In this study, we performed multi-wavelength (532, 785, 1064 nm) Raman in vitro analysis of healthy skin, BCC and SCC. Upon unified baseline correction and smoothing procedures, the acquired Raman spectra appeared rather similarly, with their main strong bands corresponding to different vibrational modes of membrane lipids, chosen for the first time as biomarkers. Their intensity ratios demonstrated distinct differentiation between healthy skin and malignant skin tumors. The MCR-ALS analysis of the Raman/PL spectra of skin neoplasms at all wavelengths confirmed the correct selection of the spectral bands for two novel wavelength-independent spectral criteria, indicating a decisive contribution of cell membrane lipids (in particular, triolein) as biomarkers.

Specifically, using the excitation wavelength of 532 nm, we achieved the reliable recognition of normal skin, BCC and SCC, for which the classification rates were 96.2%, 77.8% and 90%, respectively. The sensitivity (96.2%) and specificity (94.7%) in detecting all tumors (versus normal skin) were similar to the results obtained by dermatology experts in tumor differentiation and other methods (thermometry, ultrasound skin scanning, cross-polarization optical coherence tomography, spectroscopy and visualization in the terahertz range).

At the 785 nm excitation wavelength, the results of our classification indicated that SCC can be unmistakably distinguished from normal skin and BCC (100% classification rate). However, it is almost impossible to separate normal skin from BCC (true positive rate for BCC was 47.1%), since the intensity ratio of their Raman bands is very similar. The sensitivity and specificity at this wavelength were 84% and 72.7%, respectively.

Finally, at the 1064 nm excitation wavelength, it is possible to separate all data into only two classes, normal skin and tumors, with sensitivity and specificity of 89.5% and 96.9%, respectively. This is because BCC and SCC have no significant differences in the main bands of lipids in their Raman spectra.

Thus, the latter wavelength provided accurate in vitro differentiation between normal skin and tumors, while the 532 nm and 785 nm excitation enabled the reliable recognition of BCC and SCC. Further development of the proposed method towards the joint analysis of multi-wavelength Raman data will help to overcome the disadvantages of using each excitation wavelength separately and increase the detection rate for malignant skin tumors.

## Figures and Tables

**Table 1:** Peak positions of main Raman bands in the Raman active components.

Raman Peaks, cm^−1^	Band Assignments	Components
855	ring breathing, ν(CC)	proteins (Tyr), collagen (Pro)
936	ν(CC)	proteins (α-helix), collagen (Pro)
1002	ring breathing, ρ(CH_3_)	proteins (Phe), collagen, elastin, keratin
1078	ν(CC), skeletal stretching	triolein
1130	ν(CC), ν(CN)	lipids, proteins, ceramide
1248	Amide III	proteins (α-helix), collagen, elastin
1302	τ(CH_2_, CH_3_)	lipids, proteins (aliphatic amino acids), triolein
1336	ω(CH_2_, CH_3_)	lipids, proteins (aliphatic amino acids, Trp), elastin
1360	ν_4_–ν (Pyr half-ring)_sym_	Fermi resonance doublet of tryptophan
1442	δ(CH_2_), δ(CH_3_)	lipids, proteins (aliphatic amino acids), triolein
1450	C–H bending of proteins	proteins, keratin
1510	ν(C= C)	carotenoids
1550	ν_37_–ν(C_α_C_m_)_asym_	tryptophan
1585	ν_37_–ν(C_α_C_m_)_asym_	proteins (Phe)
1655	Amide I, ν(C=C)	proteins (α-helix), lipids (unsaturated fatty acids), elastin in proteins, triolein
1745	ν(C=O)	lipids (esters)

## Data Availability

The data supporting the reported results can be obtained from the authors.
